# Successful treatment of *Batrachochytrium salamandrivorans* infections in salamanders requires synergy between voriconazole, polymyxin E and temperature

**DOI:** 10.1038/srep11788

**Published:** 2015-06-30

**Authors:** M. Blooi, F. Pasmans, L. Rouffaer, F. Haesebrouck, F. Vercammen, A. Martel

**Affiliations:** 1 Department of Pathology, Bacteriology and Avian Diseases, Faculty of Veterinary Medicine, Ghent University, Salisburylaan 133, 9820 Merelbeke, Belgium; 2 Centre for Research and Conservation, Royal Zoological Society of Antwerp, Koningin Astridplein 26, 2018 Antwerp, Belgium

## Abstract

Chytridiomycosis caused by the chytrid fungus *Batrachochytrium salamandrivorans (Bsal)* poses a serious threat to urodelan diversity worldwide. Antimycotic treatment of this disease using protocols developed for the related fungus *Batrachochytrium dendrobatidis* (*Bd*), results in therapeutic failure. Here, we reveal that this therapeutic failure is partly due to different minimum inhibitory concentrations (MICs) of antimycotics against *Bsal* and *Bd*. *In vitro* growth inhibition of *Bsal* occurs after exposure to voriconazole, polymyxin E, itraconazole and terbinafine but not to florfenicol. Synergistic effects between polymyxin E and voriconazole or itraconazole significantly decreased the combined MICs necessary to inhibit *Bsal* growth. Topical treatment of infected fire salamanders (*Salamandra salamandra*), with voriconazole or itraconazole alone (12.5 μg/ml and 0.6 μg/ml respectively) or in combination with polymyxin E (2000 IU/ml) at an ambient temperature of 15 °C during 10 days decreased fungal loads but did not clear *Bsal* infections. However, topical treatment of *Bsal* infected animals with a combination of polymyxin E (2000 IU/ml) and voriconazole (12.5 μg/ml) at an ambient temperature of 20 °C resulted in clearance of *Bsal* infections. This treatment protocol was validated in 12 fire salamanders infected with *Bsal* during a field outbreak and resulted in clearance of infection in all animals.

The rate at which amphibian populations have been declining the past decades is alarming^1^,^2^. One of the factors in part responsible for these declines is the infectious disease chytridiomycosis caused by the chytrid fungus *Batrachochytrium dendrobatidis* (*Bd*)^3^,^4^,^5^. Recently, the related chytrid fungus *Batrachochytrium salamandrivorans* (*Bsal*) has been identified as a novel threat to amphibian populations, with a potentially major impact on salamander diversity worldwide^6^,^7^. For amphibian chytridiomycosis caused by *Bd*, topical antimycotic treatment using voriconazole at a concentration of 1.25 μg/ml during 7 days has proven highly successful and safe^8^. However, applying this treatment to *Bsal* infected salamanders is unable to clear infections (see Case report section). Thermal treatment consisting of exposure to the critical thermal maximum for *Bsal* (25 °C) for 10 days was shown to be able to clear *Bsal* infections from infected salamanders^9^. However, this temperature approaches the critical thermal maximum of several urodelans^10^, rendering this treatment of limited use for those species. Therefore the aim of this study was to develop an antifungal treatment protocol able to eliminate *Bsal* in infected salamanders at temperatures below the critical thermal maximum of *Bsal*, tolerated by most salamanders.

## Case report

Thirty-nine fire salamanders (*Salamandra salamandra*) from a population in the Netherlands undergoing dramatic declines from 2008 onwards due to *Bsal*^11^ were included in an *ex situ* conservation program. Since the susceptibility of *Bsal* to antimycotics was not known, the animals were treated with voriconazole (1.25 μg/ml, topical spray, twice a day for 7 days), based on the treatment protocol used to clear *Bd* infections from amphibians^8^. Skin lesions, lethargy and inappetite did not resolve in the *Bsal* infected animals.

## Results and Discussion

### *In vitro* susceptibility of *Bsal* to antimycotic compounds

The results of the experiments to determine the MICs of the tested antimicrobials for *Bsal* are summarized in Table 1. Florfenicol was the only compound tested that was not able to limit growth or kill *Bsal* at the concentrations tested. In contrast, florfenicol is capable of limiting growth of *Bd* at concentration of 0.5–1.0 μg/ml (Muijsers *et al.* 2012). Interestingly, the inhibitory concentrations of the other compounds against *Bsal* differed noticeably from those against *Bd*. The mechanism underlying this difference remains unknown. Whereas polymyxin E did not show any inhibitory potential in *Bd* MIC tests at the concentrations used^12^, *Bsal* was inhibited by polymyxin E at a concentration of 8000 IE/ml (Table 1). Terbinafine limited *Bsal* growth at a concentration of 0.2 μg/ml, which is in accordance with its activity against *Bd* at 0.063 μg/ml^13^. Itraconazole, which is frequently used to treat amphibians infected with *Bd*, had a MIC against *Bsal* 2.5–5 times lower (0.006 μg/ml) compared to its MIC against *Bd* (0.016–0.032 μg/ml)^13^. Finally, the MIC of voriconazole for inhibiting *Bsal* growth was 10 times higher (0.125 μg/ml) than the MIC for inhibiting *Bd* (0.0125 μg/ml)^8^. This result at least partly explains the failed initial treatment of the wild fire salamanders using the voriconazole dosage for treating chytridiomycosis in amphibians infected with *Bd* (1.25 μg/ml sprays, twice a day for 7 days)^8^. The concentrations to completely kill *Bsal* cultures were all close to the MIC (Table 1, one dilution higher for all compounds).

### Synergy between polymyxin E and voriconazole or itraconazole in inhibiting *Bsal* growth

**Figure 1:**
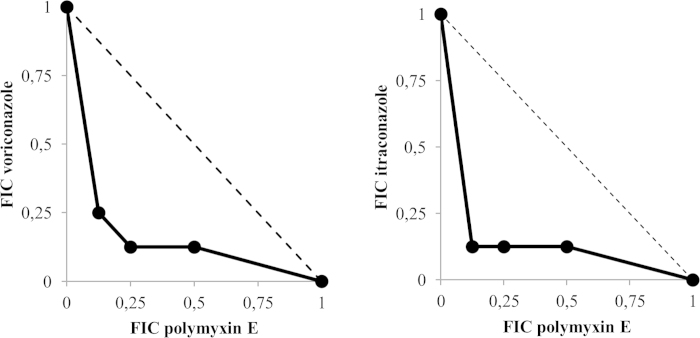
FIC Isobolograms showing the interactions between two antimicrobial agents in inhibiting *Bsal* growth. FIC values derived from combinations of voriconazole, itraconazole and polymyxin E were used to plot the isobolograms. A FIC value of 1 corresponds to the MIC value of the particular antimicrobial agent. The dotted line represents the theoretical additive interaction between two agents (see text for our definition of the types of interactions).

The three main techniques used for testing interactions between compounds in antifungal activity are Etest, time-kill methods and checkerboard dilution methods^14^,^15^,^16^,^17^. In synergy testing for bacterial pathogens, the biggest disadvantage is that no two methods will produce comparable results, and therefore clinical applicability of results is under debate^18^. These limitations also apply for antifungal synergy testing^17^. Furthermore, a vast amount of studies exist that describe *in vitro* synergy without linking (or being able to link) these results to a beneficial treatment outcome of combined treatment^18^. The goal of this study was to evaluate potential synergy between antimycotic compounds in inhibiting *Bsal* growth to allow development of an experimental treatment protocol using antifungal concentrations below toxicity levels. In this study, a checkerboard dilution method adopted from the method used to evaluate minimum inhibitory concentrations for *Bd*^8^,^12^ was used, which in comparison to the time-kill method, is easier to carry out and interpret^17^. The combinations of compounds tested both included an azole antifungal (voriconazole or itraconazole) and polymyxin E, which were already shown to be able to inhibit *Bsal* growth (Table 1). Apart from polymyxins exerting antifungal activity on their own, combinations of polymyxins and azole antifungals showed synergistic antifungal activity against infections with *Aspergillus spp*., *Candida spp*. and *Cryptococcus spp*^19^,^20^,^21^. The bactericidal activity of polymyxin E against Gram-negative bacteria is an added advantage for treating *Bsal* associated lesions, since histological preparations of skin samples of salamanders infected with *Bsal* often revealed severe bacterial overgrowth of the skin in concordance with *Bsal* infection^6^. Secondary bacterial infections in immunocompromised amphibians are often caused by opportunistic Gram-negative bacteria^22^,^23^. The azole antifungals voriconazole and itraconazole both have reported effectiveness in treating chytridiomycosis in amphibians caused by infections with *Bd*^8^,^24^,^25^. Negative side effects of treating amphibians with itraconazole have been reported though^24^,^25^,^26^, so studies evaluating the efficacy of reduced concentrations of itraconazole alone^27^ or in combination therapies to successfully treat chytridiomycosis could be a major advantage. The results of the experiments to determine the FICs of polymyxin E combined with voriconazole or itraconazole are graphically depicted in isobolograms (Fig. 1). Two of the tested combinations of polymyxin E with voriconazole (2000 IE/ml + 0.02 μg/ml and 1000 IE/ml + 0.03 μg/ml) and two combinations of polymyxin E with itraconazole (2000 IE/ml + 0.0016 μg/ml and 1000 IE/ml + 0.0016 μg/ml) resulted in a FICI that demonstrates synergism (FICI ≤ 0.5, Fig. 1). All combinations that inhibited *Bsal* growth also killed *Bsal* completely after 10 days of incubation.

### Effective treatment of *Bsal* infections in fire salamanders based on synergy between polymyxin E, voriconazole and temperature

**Figure 2:**
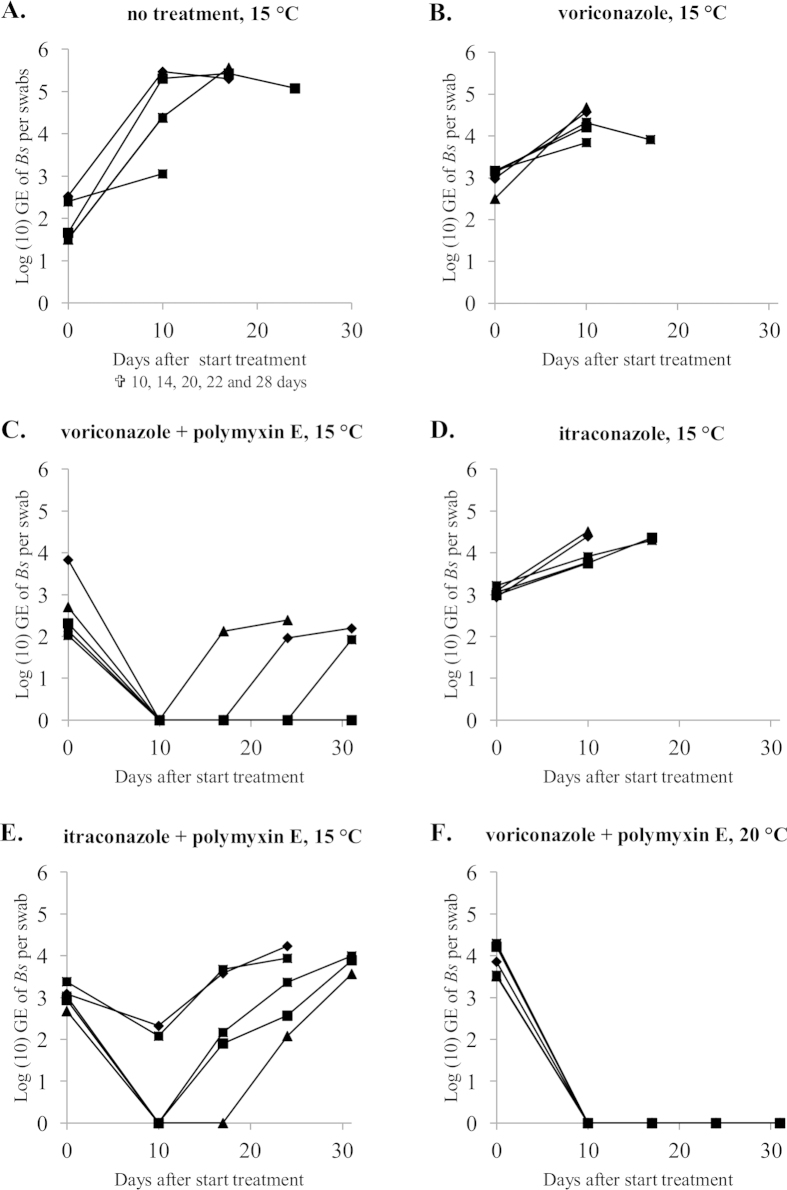
Results of five treatment protocols to clear *Bsal* infections from experimentally infected fire salamanders. After ascertaining presence of *Bsal* in all animals (day 0) they were either left untreated as control (**A**), treated twice a day for 10 days with voriconazole sprays (12.5 μg/ml) (**B**) or itraconazole sprays (0.6 μg/ml) (**D**) alone or with a combination of polymyxin E submersion baths (2000 IU/ml, 10 minutes) followed by spraying voriconazole (12.5 μg/ml) (**C**) or itraconazole (0.6 μg/ml) (**E**). Conditions A-E were all performed at an ambient temperature of 15 °C. An additional treatment protocol composed of polymyxin E submersion baths (2000 IU/ml, 10 minutes) followed by spraying voriconazole (12.5 μg/ml) at an ambient temperature of 20 °C was instituted after failure to clear *Bsal* infections in conditions A-E. Swabs for *Bsal* real-time PCR analysis were collected at day 0, 10, 17, 24 and 31. Animals that reached the determined experimental endpoint. Each symbol represents the course of infection of an individual animal.

**Figure 3:**
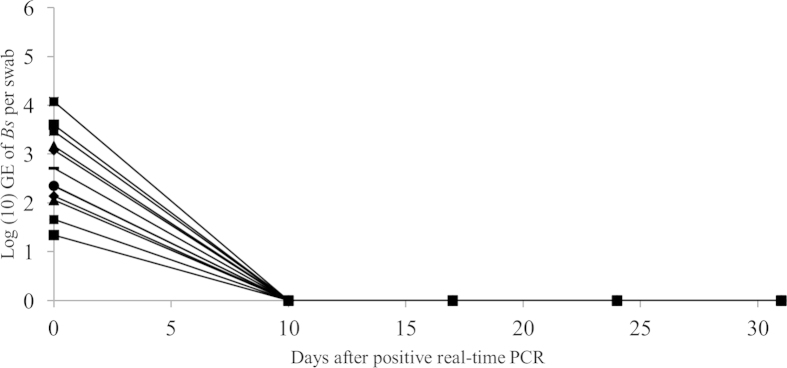
Treatment of fire salamanders naturally infected with *Bsal*. After ascertaining presence of *Bsal* in all animals (day 0), they were treated with polymyxin E submersion baths (2000 IE/ml, 10 minutes) followed by spraying voriconazole (12.5 μg/ml) twice a day for 10 days at an ambient temperature of 20 °C. Each symbol represents the course of infection of an individual animal.

All initially tested treatment conditions, composed out of itraconazole or voriconazole alone and in combination with polymyxin E were unable to clear *Bsal* infections from infected amphibians (Fig. 2, panels A–E ). Although the combination therapies of itraconazole or voriconazole with polymyxin E did reduce *Bsal* infection loads to undetectable levels in 3 out of the 5 animals and 5 out of 5 animals respectively, recrudescence of infection did occur in all animals (Fig. 2, panels C and E). The difference between *in vitro* and *in vivo* effects of the combination therapy at 15 °C might lie in the exposure to the compounds; in the *in vitro* experiments, *Bsal* was exposed continuously to both compounds as opposed to the periodical exposure in the *in vivo* experiments. At least for polymyxin E (2000 IU/ml) longer exposure times are unusable due to occurrence of toxicity (personal observations). The conditions of the additional treatment (Fig. 2, panel F) instituted after failure of the initial conditions to clear *Bsal* infections, were based on the *in vitro* synergy between voriconazole and polymyxin E in inhibiting *Bsal* growth, the increased but suboptimal inhibition of *Bsal in vivo* by combined exposure to voriconazole and polymyxin E (Fig. 2, panel C) and the previously determined temperature dependent infection dynamics of *Bsal*^9^. Using the same concentrations of voriconazole and polymyxin E, but raising the temperature to 20 °C did result in successful elimination of *Bsal* in all infected animals (Fig. 2, panel F). The results of this study underline the key influence temperature plays in *Bsal* infection dynamics, which already allowed development of a *Bsal* temperature treatment protocol for amphibian species able to endure a continuous ambient temperature of 25 °C for 10 days^9^. Ethical considerations allowed only one additional treatment condition to be tested. Therefore, the positive treatment effect could theoretically be attributed to the sole influence of either one of the compounds at 20 °C, as experimental treatments with individual compounds were only tested *in vivo* at a temperature of 15 °C. The results of this study show that synergy between voriconazole and polymyxin E together with the temperature dependent infection dynamics of *Bsal* allow *Bsal* infections to be eliminated in amphibian species with critical thermal maxima lower than that of *Bsal*. The efficacy of the treatment protocol was validated by successful treatment of fire salamanders naturally infected with *Bsal* during a field outbreak (Fig. 3). In conclusion, *in vitro* synergy between antimycotic compounds in inhibiting *Bsal*, together with the temperature dependent infection dynamics of *Bsal* allowed development of a treatment protocol successful in eliminating *Bsal* from experimentally and naturally infected amphibians. Although exploiting synergism between temperature and chemical compounds was effective in this study, in case of therapeutic failure, acclimatisation of *Bsal* to higher temperatures and/or the emergence of *Bsal* strains with higher thermal preferences should be considered^28^,^29^.

## Methods

All experiments were performed in accordance with the relevant guidelines and regulations. All experiments with experimental animals were carried out with approval of the ethical committee of the Faculty of Veterinary Medicine, Ghent University.

### Chytrid strain & culture conditions

The *Bsal* type strain (AMFP13/1)^6^ was grown in TGhL broth (16 g tryptone, 4 g gelatin hydrolysate, 2 g lactose per liter of distilled water) in 25 cm^3^ cell culture flasks and incubated at 15 °C. To obtain a suspension containing a mixture of zoosporangia and zoospores, the walls of a cell culture flask containing 5-day-old culture were scraped with a sterile cell scraper and the suspension subsequently collected.

### Determination of the minimum inhibitory concentrations of antimicrobial agents against *Bsal*

The minimum inhibitory concentrations (MICs) of florfenicol (20%), voriconazole (VFend IV, Pfizer, Kent, UK), itraconazole (Itrafungol, Elanco, Brussels, Belgium), terbinafine (Terbinafine hydrochloride, Sigma-Aldrich, Bornem, Belgium) and polymyxin E (Colistin sulphate, VMD, Arendonk, Belgium) for *Bsal* were determined using a broth macrodilution method used for MIC testing of *Bd*^8^,^12^. In short, two-fold dilutions series of the antimicrobial agents were prepared in TGhL broth, and 200 μl of these prepared dilutions were added to wells of 24 well cell culture plates (Table 1). Two hundred μl of a suspension containing a mixture of *Bsal* sporangia and zoospores (approximately 10^5^ *Bsal* organisms per ml) were added to all wells. Finally, 1600 μl of TGhL broth were added to all wells resulting in a final volume of 2 ml per well. Plates were incubated at 15 °C (optimum growth temperature of *Bsal*^6^) and checked for viability and growth daily for 10 days with an inverted light microscope. Wells containing TGhL broth with viable *Bsal* sporangia and zoospores and wells containing heat treated (85 °C, 10 minutes) *Bsal* sporangia and zoospores served as positive and negative growth controls respectively. The MIC value was determined as the lowest concentration of the antimicrobial agent at which no growth could be observed after 10 days of incubation. To test which concentrations of the antimicrobial agents were lethal for *Bsal* after 10 days of exposure, we removed the medium and replenished all wells with fresh TGhL broth without antimicrobial agents. Plates were incubated at 15 °C and checked for viability and growth daily for an additional 14 days with an inverted light microscope. A concentration was considered to be lethal to *Bsal* when no signs of growth could be observed after this incubation period of 14 days (Table 1). All conditions were tested in triplicate.

### Determination of the fractional inhibitory concentrations of antimicrobial agents against *Bsal*

To test for synergy in combinations of polymyxin E with voriconazole or with itraconazole in inhibiting *Bsal* growth, fractional inhibitory concentrations (FICs)^30^ were determined using a macrodilution broth checkerboard technique. Two-fold serial dilution series of all antimicrobial agents in TGhL broth were prepared. Polymyxin E was tested at final concentrations of 1000–64000 IE/ml, voriconazole at final concentrations of 0.016–1 μg/ml and itraconazole at final concentrations of 0.0007–0.05 μg/ml. All tested concentrations of the individual antimicrobial agents were included separately as controls for reproducibility of the earlier determined MIC values of the compounds. Twenty-four well cell culture plates were prepared with 1600 μl of TGhL broth, 200 μl of the respective compound or combination of compounds and 200 μl of a suspension containing *Bsal* sporangia and zoospores including *Bsal* positive and negative growth controls as described earlier. Plates were incubated at 15 °C and checked for signs of viability and growth daily for 10 days with an inverted light microscope. The FIC value for an individual antimicrobial agent is determined as the ratio of the MIC value of the antimicrobial agent used in combination (MIC_combi_) to the MIC value of the antimicrobial by itself (MIC_alone_) after 10 days of incubation:


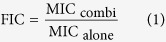


These FIC values are subsequently used to produce a single fractional inhibitory concentration index (FICI) as an indicator for the type of interaction between two antimicrobial agents^30^:


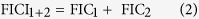


We determined the possible interactions as synergistic (FICI ≤ 0.5), additive (FICI > 0.5–1.0), indifferent (FICI > 1.0–4.0) or antagonistic (FICI ≥ 4.0)^18^,^31^,^32^. Isobolograms were used to graphically depict the FIC and FICI values of all tested combinations of antimicrobial agents (Fig. 1)^33^. To test which combination of concentrations of the antimicrobial agents was lethal for *Bsal* after 10 days of exposure, we replaced the broth containing the compound(s) with fresh TGhL broth without antimicrobial agents. Plates were incubated at 15 °C and checked for viability and growth daily for an additional 14 days with an inverted light microscope. A combination of concentrations was considered to be lethal to *Bsal* when no signs of growth could be observed after this incubation period of 14 days. All conditions were tested in quadruplicate.

### Treatment of experimentally infected fire salamanders

Fire salamanders (*Salamandra salamandra*) were inoculated with *Bsal* in order to study *in vivo* efficacy of different antimicrobial treatment protocols. The animal experiment was performed with the approval of the ethical committee of the Faculty of Veterinary Medicine (Ghent University, EC2013/87 and EC2014/65). Thirty captive bred fire salamanders were housed individually in plastic containers in a climatized room with an ambient temperature of 15 °C. The animals were kept on a moist tissue, with access to a hiding place and water container. Crickets powdered with mineral and vitamin supplement were provided ad libitum as food source. All animals were clinically healthy and free of *Bd* and *Bsal*, as determined with duplex real-time PCR examination of skin swabs^34^. An acclimatization period of 2 weeks was admitted before the start of the experiment. The experimental animals were randomly assigned to one of the 6 experimental treatment groups (5 animals per treatment group, kept individually). All salamanders were inoculated with *Bsal* by topically applying one mL of inoculum containing 10^5^ zoospores per ml on the skin. Animals were kept at 15 °C (except for the animals in group F; details below) and skin swabs for *Bsal* real-time PCR analysis^34^ were collected every 7 days. Individual treatment commenced when *Bsal* infection was established (determined as an increase in *Bsal* infection load between 2 consecutive samplings). The different groups were untreated negative control (group A), voriconazole treatment (12.5 μg/ml) alone (group B), voriconazole and polymyxin E treatment (concentrations of 12.5 μg/ml and 2000 IU/ml respectively, group C), itraconazole treatment (0.6 μg/ml) alone (group D) and itraconazole and polymyxin E treatment (0.6 μg/ml and 2000 IU/ml respectively, group E). After initial failure to clear *Bsal* infections in these first experimental groups, we trialed another treatment condition composed of voriconazole and polymyxin E treatment (concentrations of 12.5 μg/ml and 2000 IU/ml respectively, identical to the treatment described for group C) but with an ambient temperature of 20 °C instead of 15 °C (group F). Due to ethical considerations only one additional condition was performed. All experimental treatments were carried out twice a day for 10 days. Polymyxin E was administered through submersion baths (10 minutes) and voriconazole and itraconazole were administered through spraying the animals and tissue in their housing (after polymyxin E baths if applicable). After the treatment period, all animals were kept at 15 °C. Skin swabs for *Bsal* real-time PCR analysis were collected immediately after the treatment period and subsequently every 7 days for another 3 weeks. An animal was considered negative for *Bsal* after 3 consecutive negative real-time PCR results. Development/progression of symptoms associated with *Bsal* infections together with presence of *Bsal* as determined with real-time PCR analysis in an animal was determined as experimental endpoint and resulted in withdrawal of the animal from the experiment. If an animal tested positive for the presence of *Bsal* in the post-treatment follow-up phase (starting from day 10 in Fig. 2), the treatment was considered as failed. Remaining *Bsal* infections in animals that were removed from the experiment due to reaching the described endpoint, and animals still positive for *Bsal* at the last sampling time point were exposed to an ambient temperature of 25 °C during 10 days to clear the *Bsal* infection^9^.

### Treatment of naturally infected fire salamanders

Thirty-five fire salamanders from the population from which *Bsal* (strain AMFP13/1) was originally isolated (Bunderbos, Netherlands, N50°54′51″, E5°44′59″) were transferred to our research facility for treatment. Upon arrival, 12 of the translocated animals tested positive for presence of *Bsal* DNA as tested with the *Bsal* real-time PCR^34^. Based on the results of the treatments of experimental infections, the animals were treated with polymyxin E submersion baths (2000 IU/ml, 10 minutes) followed by spraying voriconazole (12.5 μg/ml) twice a day for 10 days at an ambient temperature of 20 °C. Housing conditions of the animals were identical to the conditions described for the experimental animals. After the treatment period all animals were put back at 15 °C. Skin swabs for *Bsal* real-time PCR analysis were collected directly after the treatment period and subsequently every 7 days for another 3 weeks. An animal was considered negative for *Bsal* after 3 consecutive negative real-time PCR results.

### Cost of treatment

A step-by-step treatment protocol can be found as supplementary file. Based on the products, volumes and estimated pricing in this protocol, the treatment cost of a single animal would be 6 €. It should be noted however, that this estimated cost applies for individual treatment of animals. Treatment of groups of amphibians with the same volumes is possible (although this has not yet been validated), resulting in a reduction of the cost of treatment per animal.

## Additional Information

**How to cite this article**: Blooi, M. *et al.* Successful treatment of *Batrachochytrium salamandrivorans* infections in salamanders requires synergy between voriconazole, polymyxin E and temperature. *Sci. Rep.* **5**, 11788; doi: 10.1038/srep11788 (2015).

## Supplementary Material

Supplementary Information

## Figures and Tables

**Table 1:** Susceptibility of *Bsal* to antimicrobial compounds.

Compound	2-fold dilution range	MIC value	100% killing concentration
Florfenicol	0.016–8 μg/ml	>8 μg/ml	>8 μg/ml
Voriconazole	0.016–8 μg/ml	0.125 μg/ml	0.25 μg/ml
Polymyxin E	1250–64000 IE/ml	8000 IE/ml	16000 IE/ml
Itraconazole	0.003–1.2 μg/ml	0.006 μg/ml	0.012 μg/ml
Terbinafine	0.1–12.5 μg/ml	0.2 μg/ml	0.4 μg/ml

An overview of the tested two-fold serial dilution range of the antimicrobial compounds, the minimum inhibitory concentrations (MICs) for *Bsal* and the concentrations that completely killed *Bsal* after an exposure period of 10 days.
