# Supplementary material for: Successful treatment of Batrachochytrium salamandrivorans infections in salamanders requires synergy between voriconazole, polymyxin E and temperature
Source: Sci Rep. 2015 Jun 30;5:11788. doi: 10.1038/srep11788 (PMC4485233; doi:10.1038/srep11788)
Supplement: Supplementary Information [file srep11788-s1.pdf]

Supplementary files for:

Successful treatment of *Batrachochytrium salamandrivorans* infections in salamanders requires synergy between voriconazole, polymyxin E and temperature

M. Blooi*, F. Pasmans, L. Rouffaer, F. Haesebrouck, F. Vercammen, A. Martel

*corresponding author. Email: Mark.Blooi@UGent.be

This PDF file includes:

Step-by-step treatment protocol
Supplementary references

General considerations

- All disposable waste materials (gloves, tissues, water, etc.) should be disinfected before being discarded.
- Wear powder-free nitrile gloves when handling animals, and change gloves in-between animals.
- House and treat *Bsal* infected animals in quarantine facilities.
- Store stock and working solutions of voriconazole and polymyxin E at 4 °C.

Preparing stock and working solutions

Polymyxin E, suggested formulation: Colistin sulphate 1000000 IU/ml, 40 ml, VMD, Arendonk, Belgium (approximate price 10 €).
Dilute stock solution in distilled water to achieve a working concentration of 2000 IU/ml polymyxin E. Four liters of polymyxin E (2000 IU/ml) are sufficient for the complete treatment of 1 amphibian (based on using 200 ml of solution per polymyxin E bath; changes to this volume might apply depending on the size of the bathing container and amphibian species being treated). As degradation of polymyxin E in aqueous solution might occur[S1], we recommend preparing fresh working solutions of polymyxin E each day .

Voriconazole, suggested formulation: VFend IV 200 mg, Pfizer, Kent, UK (approximate price 160 €).
Prepare to the manufacturer's instructions to achieve a solution of 10 mg/ml voriconazole.
Dilute stock solution in distilled water to achieve a working concentration of 12.5 µg/ml voriconazole. 0.4 liter of voriconazole (12.5 µg/ml) is sufficient for the complete treatment of 1 amphibian (based on using 20 ml of solution per voriconazole spray treatment). Voriconazole has been proven to be stable in solution and stock solution can be stored during the duration of the treatment[S2.]. The use of the less expensive oral formulation can result in tadpole toxicity (unpublished results).

Treatment protocol

It is imperative to keep animals at 20 °C during the complete treatment period!

Prepare stock and working solutions as described. Store stock and working solutions at 4 °C.

The treatment consists of two steps:

1. Transfer the animal into a container containing the polymyxin E solution (2000 IU/ml). The animal should be submerged in the solution, with just its head above the surface. Bathe the animal in the solution for 10 minutes. Rinse the animal after the bath with distilled water. Discard the polymyxin E solution, and take care to completely wipe dry the container (one bathing container can be used throughout the complete treatment of a single animal).

2. Transfer the animal to a quarantine terrarium with dry tissue on the bottom. Spray approximately 20 ml of voriconazole solution (12.5 µg/ml) on the animal and tissue using a sprayer (any sprayer able to create a fine mist will do).

Animals should be treated twice per day during 10 days. Depending on the size of the animals and the containers used, amphibians can be treated in groups, which reduces the amount of medication used. After completing the treatment, place the animal back at its preferred temperature and consider the animal as potentially *Bsal* positive until proven negative. An animal is considered negative for *Bsal* after 3 consecutive negative real-time PCR results of skin swabs, collected at one week intervals. The collection of skin swabs and the real-time PCR have been described previously[S3, S4]. Should a positive real-time PCR result be obtained, repeat the complete treatment.

Supplementary references

S1. Orwa, J.A. *et al.* Study of the stability of polymyxins B_1, E_1 and E_2 in aqueous solution using liquid chromatography and mass spectrometry. *J. Pharm. Biomed. Anal.* **29**, 203-212 (2002).

S2. Martel, A. *et al.* Developing a safe antifungal treatment protocol to eliminate *Batrachochytrium dendrobatidis* from amphibians. *Med. Myc.* **49**, 143-149 (2011).

S3.Hyatt, A.D. *et al.* Diagnostic assays and sampling protocols for the detection of *Batrachochytrium dendrobatidis*. *Dis. Aquat. Organ.* **73**, 175-192 (2007).

S4. Blooi, M. *et al.* Treatment of urodelans based on temperature dependent infection dynamics of *Batrachochytrium salamandrivorans*. *Sci Rep.* **5**, 8037 (2015).
